# Lateralization in the Invertebrate Brain: Left-Right Asymmetry of Olfaction in Bumble Bee, *Bombus terrestris*

**DOI:** 10.1371/journal.pone.0018903

**Published:** 2011-04-27

**Authors:** Gianfranco Anfora, Elisa Rigosi, Elisa Frasnelli, Vincenza Ruga, Federica Trona, Giorgio Vallortigara

**Affiliations:** 1 Research and Innovation Centre, Fondazione Edmund Mach, San Michele all'Adige, Italy; 2 Centre for Mind/Brain Sciences, University of Trento, Rovereto, Italy; University of Queensland, Australia

## Abstract

Brain and behavioural lateralization at the population level has been recently hypothesized to have evolved under social selective pressures as a strategy to optimize coordination among asymmetrical individuals. Evidence for this hypothesis have been collected in Hymenoptera: eusocial honey bees showed olfactory lateralization at the population level, whereas solitary mason bees only showed individual-level olfactory lateralization. Here we investigated lateralization of odour detection and learning in the bumble bee, *Bombus terrestris* L., an annual eusocial species of Hymenoptera. By training bumble bees on the proboscis extension reflex paradigm with only one antenna in use, we provided the very first evidence of asymmetrical performance favouring the right antenna in responding to learned odours in this species. Electroantennographic responses did not reveal significant antennal asymmetries in odour detection, whereas morphological counting of olfactory sensilla showed a predominance in the number of olfactory sensilla trichodea type A in the right antenna. The occurrence of a population level asymmetry in olfactory learning of bumble bee provides new information on the relationship between social behaviour and the evolution of population-level asymmetries in animals.

## Introduction

Research on anatomical and functional side-related specializations of the brain has mainly focused on vertebrates [1], [2], [3]. Recently, evidence for brain and behavioural lateralization among invertebrates has been reported [4], [5], [6], [7], [8], [9], [10].

Olfactory asymmetries have been shown in honey bees by Letzkus et al. [11]. When conditioned, using the proboscis extension reflex paradigm (PER) [12], with only one antenna in use, bees showed better learning with their right rather than their left antenna. Evidence for lateralization of olfactory learning in the honey bee has been subsequently confirmed and extended exploiting the same paradigm, using different scents and without any coating of the antennae, i.e. with lateral presentations of odour stimuli [13], [14], [15], [16].

In natural conditions, asymmetries may occur at the population-level when more than 50% of the individuals are lateralized in the same direction, whereas lateralization at the individual level occurs when most of the individuals are lateralized with either a left- or right- bias equally distributed in the population [1]. Recently, the issue has been tackled of the advantages for an individual in a population of being bound into directional behavioural asymmetries [2]. Ghirlanda and Vallortigara [17] showed, using mathematical game theory, that in a prey-predator ecological context, population-level lateralization might represent an evolutionary stable strategy (ESS) driven by social pressures, i.e. when asymmetrical organisms have to coordinate their asymmetries in behaviour among each other. A well-fitting example might be the turning behaviour to escape from a predator in shoaling fish species. In a large number of teleost fishes the shoaling species appeared to be lateralized at the population level, while the majority of non-shoaling species were lateralized at the individual level [18].

Strictly related species of bees (Superfam. Apoidea) with different levels of intraspecific social interactions may provide important evidence in order to evaluate the hypothesis that population-level asymmetries are more likely to occur among social species. Anfora et al. [14] recently reported that two different species of bees, *Apis mellifera*, the most sophisticated eusocial species, and *Osmia cornuta*, a solitary species, showed different olfactory asymmetry behaviours. The eusocial species appeared to be lateralized at the population level, whereas the solitary species appeared to be lateralized only at the individual level.

Here we studied an annual social species of Apoidea, *Bombus terrestris* L. (Hymenoptera: Apidae). These bumble bees exhibit primitive eusocial behaviour as they have an annual cycle with single queens founding new annual nests. Therefore, bumble bees can represent one of the last evolutionary steps in the taxonomic group of Hymenoptera towards the complete development of eusociality [19], [20]. We tested olfactory learning in bumble bees with only one antenna in use. Given that in honey bees behavioural lateralization in olfactory learning has been associated with anatomical and electrophysiological asymmetries at the peripheral level in the olfactory neural pathway [11], [14], [16], we also measured the number of putative olfactory sensilla in the left and the right antennae using scanning electron microscopy and the electrophysiological responses of the two antennae when stimulated by odours behaviourally relevant to bumble bees.

## Materials and Methods

### Insects

For all the experiments bumble bee foragers were collected from the same colony of *B. terrestris*, supplied by Bioplanet s.c.a., Cesena, Italy. The tested individuals were not age-marked but they could be considered to have had similar olfactory experiences because of prior exposition to the same odours inside the colony and because they were not allowed to forage outside the nest.

We used female foragers of similar size (mean body size: 1.7 cm) in order to minimize naturally occurring antennal sensitivity variations [21].

### Test compounds

The test synthetic chemicals were two odours behaviourally relevant to bumble bees: isoamylacetate (Sigma-Aldrich, Milano, Italy; >99.7% purity), both component of their pheromone blends and a floral compound, and (-)-linalool (Sigma-Aldrich, >98.5% purity), a common floral compound, [22], [23].

### Behavioural experiments

Behavioural methods made use of the experimental procedures developed in honey bees [11], [12], [13] and bumble bees [23] for olfactory learning. After 12 hours of food deprivation, bumble bees were cooled in 750 ml containers until immobilized and secured in metal holders. The insects were randomly assigned to three different groups; with the left (N = 10) or the right (N = 10) antenna coated with a two-components silicon compound (Silagum-Mono, DMG, Germany), or with both the antennae uncoated (N = 10) [11], [14]. Training started one hour after the antennae had been coated. Each animal in its holder was in turn placed in front of an exhaust fan and trained using (-)-linalool, plus 1 M sucrose solution (reward) as a positive stimulus (10 µl of (-)-linalool dissolved in 3 ml of the sugar solution). The negative stimulus was unscented saturated NaCl solution. Three learning trials were given every 6 min. On the first trial a drop of the positive stimulus solution at the end of a 23 gauge needle was held 1 cm over the antennae and after 5 s the antennae were touched, which led to PER. The bumble bee was then allowed to ingest the drop of (-)-linalool sugar solution as reward. The procedure was immediately repeated with the saline solution, which did not trigger PER but avoidance by moving the antennae away from the negative stimulus. On the two subsequent training trials the procedure of the first trial was repeated (and usually PER occurred without the need to touch the antennae). To check possible behavioural differences among the three groups during the training procedures, we performed analysis of variance (ANOVA) with antenna in use as a between-subject factor, considering the number of proboscis extensions over the total conditioning trials.

Recall of short-term odour memory was tested 1 hour after the end of training. Both (-)-linalool, dissolved in distilled water at the same concentration as used in training, and saturated salt solution were presented holding a drop of these solutions over bumble bee's antennae for 5 s being careful not to touch them. Each animal was tested in a total of 10 such paired trials, presented in random order and separated by an inter-trial interval of 60 s. It has been demonstrated, in fact, that habituation in PER responses does not occur over 20 trials [13]. We recorded every time the bumble bee extended the proboscis. The percentage of correct responses was calculated as number of proboscis extensions to the (-)-linalool over the total (-)-linalool presentations per animal (no proboscis extensions to salt solution occurred).

Data were analyzed by analysis of variance (ANOVA) with antenna in use as a between-subjects factor.

### Electroantennography (EAG)

Absolute EAG responses (mV) were recorded from right and left isolated antennae of *B. terrestris* foragers (N = 20) with a standard EAG apparatus (Syntech, Hilversum, The Netherlands). Animals were anaesthetized, antennae were cut at the level of the scape and the uppermost part of the antennal tip was removed. The base of the antenna was placed inside a glass micropipette filled with Kaissling saline solution [24] and the tip put into the recording glass micropipette electrode. The first antenna tested was chosen randomly and the animal was kept alive until the second antenna was used.

The test synthetic compounds were isoamylacetate and (-)-linalool. For each compound, 25 µl of five decadic steps hexane solutions (ranging from 10^−2^ to 10^2^ µg/µl) were absorbed on 1 cm^2^ pieces of filter paper, inserted into individual Pasteur pipettes and put into the constant air flow tube directed to the antenna (50 cm^3^/s). Stimuli of 500 ms were presented in ascending order of dosage with 30 s inter-stimuli intervals, using a stimulus controller (CS-55, Syntech). Control pipettes (loaded with 25 µl of hexane and an empty pipette) were used before and after each series of stimuli. Data were log transformed to account for the heterogeneity of variances and analyzed by analysis of variance (ANOVA) with antenna, scent and dose as within-subject factors.

### Scanning Electron Microscopy (SEM)

**Figure 1:**
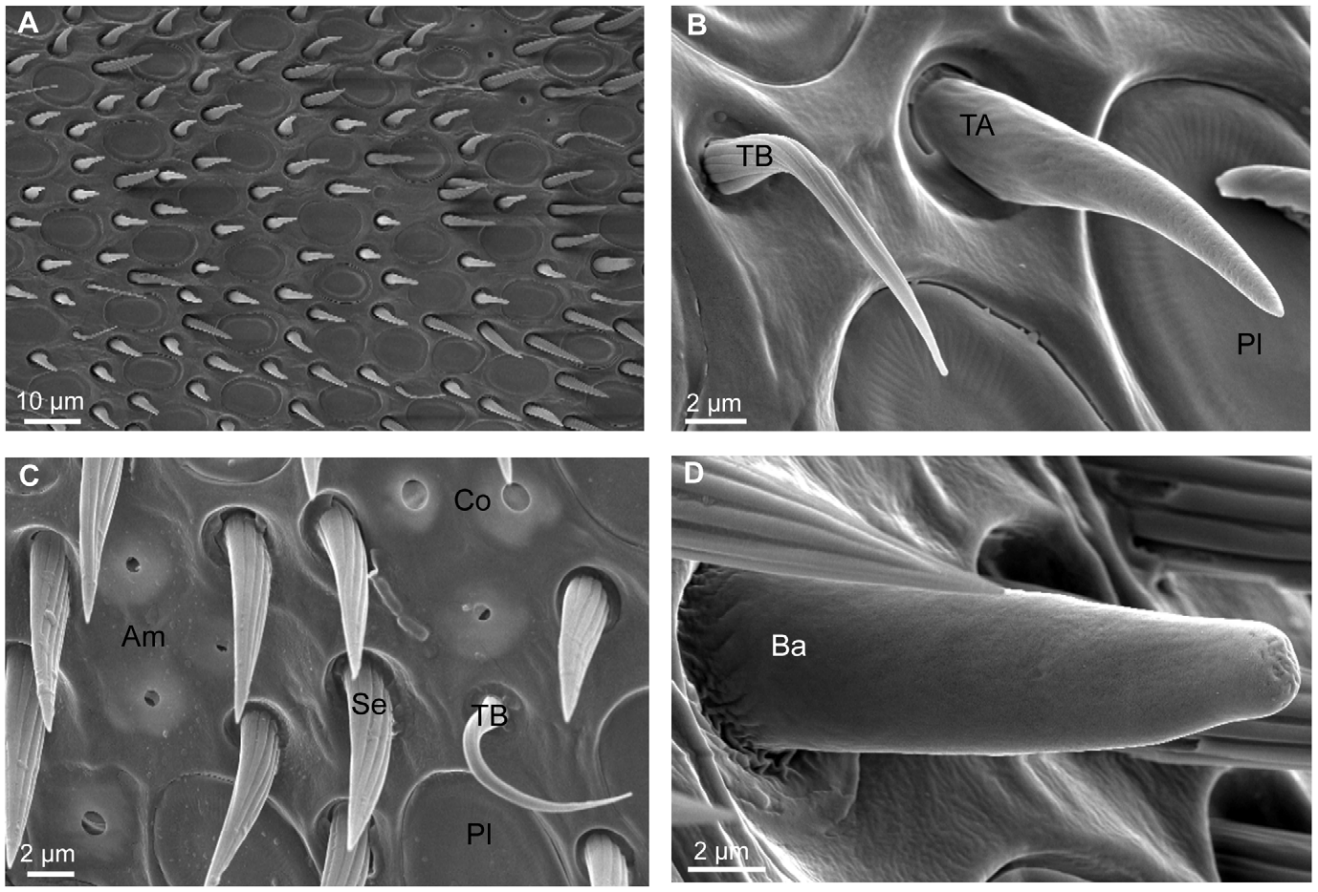
Scanning electron micrographs of *Bombus terrestris* foragers. (a) ventral view of a medial segment of the flagellum; (b) details of sensillum trichodeum type A, type B and sensillum placodeum; (c) details of sensillum coeloconicum, ampullaceum, trichodeum type B and setae; (d) detail of sensillum basiconicum. Am, sensillum ampullaceum; Ba, sensillum basiconicum; Co, sensillum coeloconicum; Pl, sensillum placodeum; Se, seta; TA, sensillum trichodeum type A; TB, sensillum trichodeum type B.

Bumble bees (N = 14) were anaesthetized and their left and right antennae were cut at the base of pedicel. The basal segments of each pair of antennae were attached to a circular stub by double-sided conductive tape (TAAB Laboratories Equipment Ltd. Aldermaston, UK). All samples were gold coated for guaranteeing electrical conductivity and scanned with a XL 30, Field Emission Environmental Scanning Electron Microscope (FEI-Philips, Eindhoven, The Netherlands). Each antenna was imaged from four different viewpoints: ventral (holder at 0°), right (sample tilted at −75°), left (sample tilted at +75°) and dorsal (after removing antenna from stub and replacing upside). Because of the lack of olfactory sensilla on the first two segments of the flagellum of *B. terrestris*, only segments from 3^rd^ to 10^th^ were scanned. Each segment from 3^rd^ to 9^th^ was scanned longitudinally at a magnification of 600 times (Figure 1a). A magnification of 800 times was used for the 10^th^ segment (apex). For each segment four images were collected according to the different viewpoints.

Both putative olfactory sensilla, i.e. sensilla placodea (Figure 1b–c), trichodea type A (Figure 1b), coeloconica (Figure 1c), and basiconica (Figure 1d), and non-olfactory sensilla, i.e. sensilla trichodea type B (Figure 1b), and ampullacea (Figure 1c), were identified according to their specific morphological characteristics as described in Frasnelli et al. [16] and in Ågren and Halberg [25]. Each type of sensillum was then tagged and counted on all acquired images by using image analysis software (UTHSCSA ImageTool Version 3.0). Data were clustered according to the four viewpoints, eight antennal segments, two antennae and six sensillum types. Data were analyzed by analysis of variance with antenna, segment and type of sensilla as within-subjects factors. Each sensillum type was analyzed by analysis of variance (ANOVA) with antenna and segment as within-subjects factors.

## Results

### Behavioural experiments

No behavioural differences emerged among the three groups during the training procedures (F_2,27_ = 1.02, p = 0.375).

**Figure 2:**
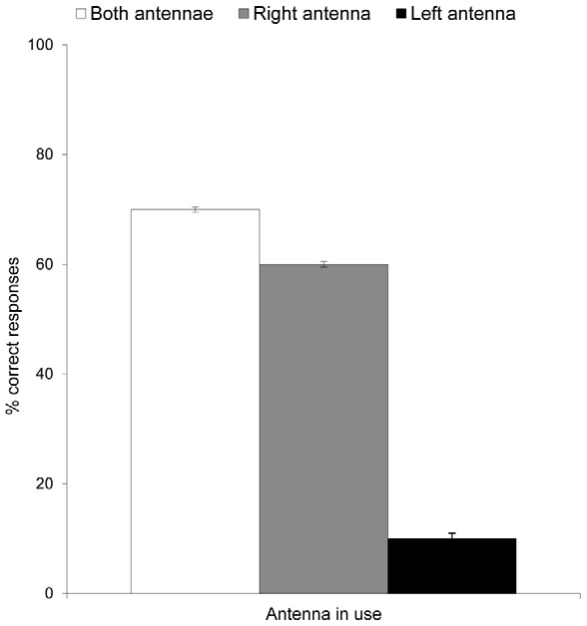
Behavioural asymmetry during recall of short-term odour memory in *Bombus terrestris* foragers, after trained on the proboscis extension reflex. Mean percent correct responses ± SE 1 h after (-)-linalool conditioning with both antennae in use (white bars), right antenna in use only (grey bars), or left antenna in use only (black bars). A significant effect of the antenna in use was found (ANOVA: F_2,27_ = 80.86, p<0.001). Post hoc comparison using Tukey HSD test revealed a significant difference between bees using their right and their left antenna (p<0.001), and between bees using their left antenna and those using both antennae (p<0.001) and between bees using their right antenna and bees using both antennae (p<0.01).

The analysis of variance of correct responses 1 h after training revealed a significant effect of the antenna in use (F_2,27_ = 80.86, p<0.001) (Figure 2). Post hoc comparison using Tukey HSD test revealed a significant difference between bees using their right and their left antenna (p<0.001), and between bees using their left antenna and those using both antennae (p<0.001) and between bees using their right antenna and bees using both antennae (p<0.01).

### Electroantennography (EAG)

**Figure 3:**
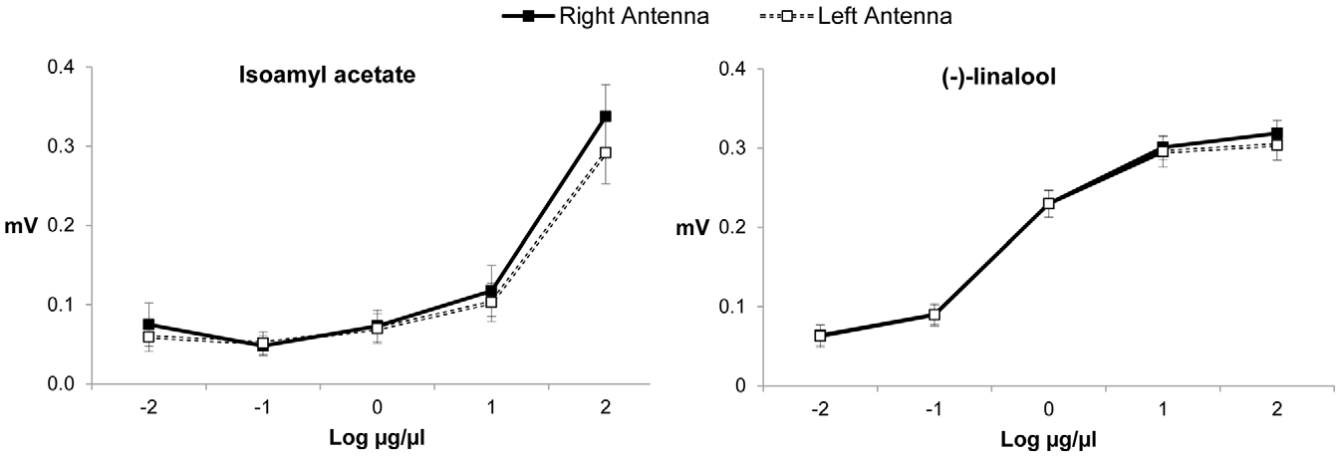
Mean EAG ± SE absolute responses (mV) of right (unbroken lines with black squares) and left (dotted lines with empty squares) antenna of *Bombus terrestris* foragers (N = 20) to isoamyl acetate (left) and (-)-linalool (right) at five different doses (Log_10_ µg/µl). No significant differences were found between the antennae (ANOVA: F_1,19_ = 2.72, p = 0.12). Significant effects of both dose (ANOVA: F_4,16_ = 42.52, p<0.001) and scent (ANOVA: F_1,76_ = 107.61, p<0.001) were revealed.

The results of electroantennography are shown in Figure 3. The EAG responses elicited by the tested odours, isoamylacetate and (-)-linalool, were not significantly different between the right and the left antenna (F_1,19_ = 2.72, p = 0.12). Though not lateralized at the population level, 12 out of 20 individual bumble bees showed significantly stronger responses (estimated by one-tailed binomial test, p<0.05) either with the right (9 animals) or the left (3 animals) antenna (one-tailed binomial test, p = 0.054). The ANOVA also revealed a significant increase in EAG responses with increasing doses of both tested odours (F_4,16_ = 42.52, p<0.001), a significant effect of the type of odours (F_1,76_ = 107.61, p<0.001) and a significant interaction between type of odours and dose (F_4,76_ = 20.49, p<0.001).

### Scanning Electron Microscopy (SEM)

**Figure 4:**
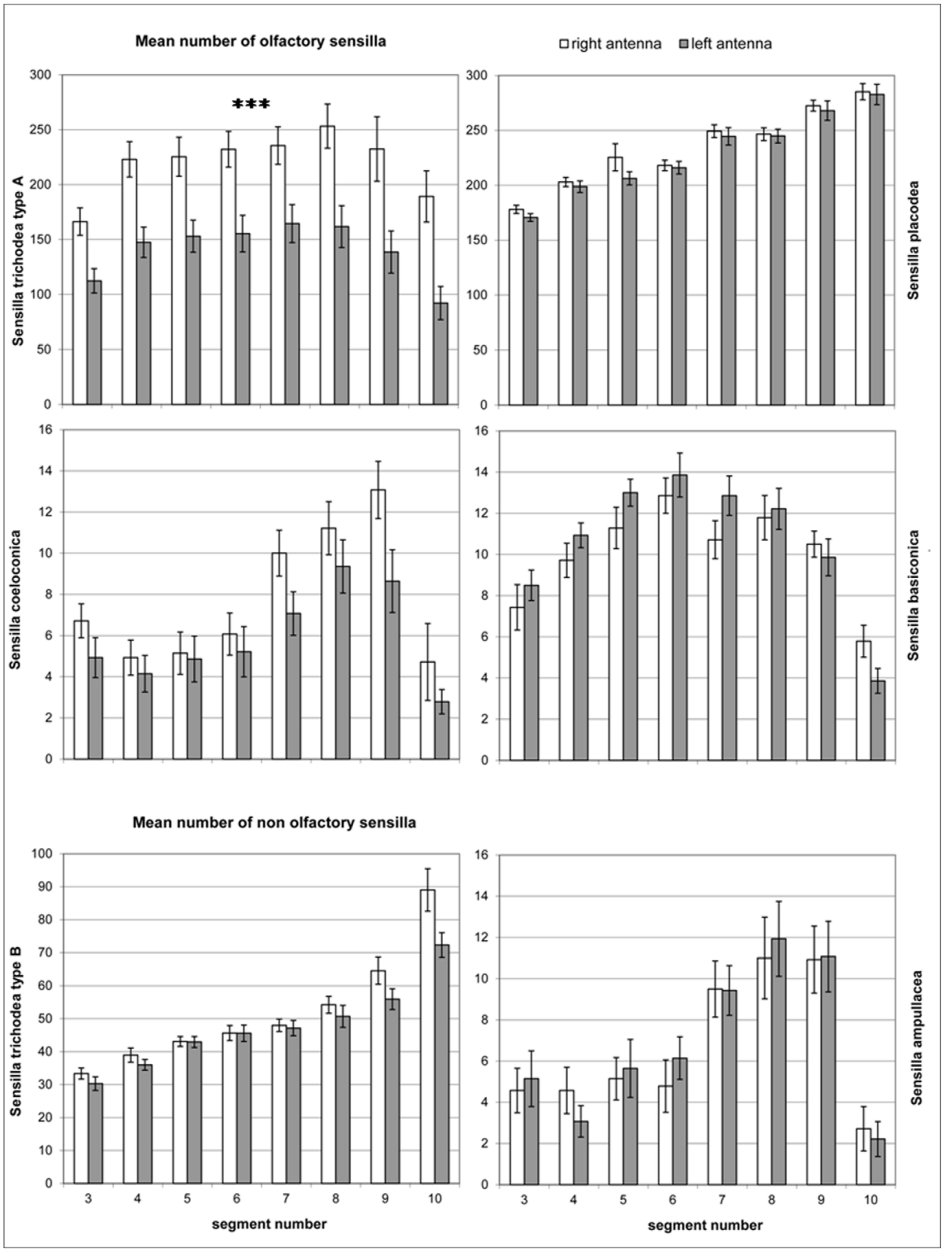
Mean number ± SE of sensilla for the right antenna (white bars) and for the left antenna (grey bars) of *Bombus terrestris* foragers in function of the segment number. Putative olfactory sensilla: placodea, trichodea type A, basiconica, coeloconica (upper graphs). Non-olfactory sensilla: trichodea type B, ampullacea (lower graphs). Data were analyzed by ANOVA with antenna, segment and sensilla as within-subjects factor. An overall antenna effect emerged (F_1,13_ = 22.56, p<0.001). A significant effect of segment (F_7,91_ = 43.20, p<0.001), sensillum type (F_5,65_ = 396.40, p<0.001) and antenna per sensillum type interaction (F_5,65_ = 17.89, p<0.001) was revealed. Asterisks indicate a significant right antenna dominance in the number of olfactory sensilla trichodea type A (F_1,13_ = 21.26, p<0.001). No significant antenna effects were found in the number of sensilla basiconica (F_1,13_ = 1.47, p = 0.247), sensilla coeloconica (F_1,13_ = 3.61, p = 0.08) and sensilla placodea (F_1,13_ = 0.97, p = 0.342). Analyses of non-olfactory sensilla did not reveal any significant difference between right and left antennae in the number of sensilla trichodea type B (F_1,13_ = 3.45, p = 0.086) and sensilla ampullacea (F_1,13_ = 0.10, p = 0.755).

The results of SEM analysis are shown in Figure 4. The overall number of sensilla analyzed appeared to be higher on the right than on the left antenna (F_1,13_ = 22.56, p<0.001). The analysis of variance also revealed significant effect of segment (F_7,91_ = 43.20, p<0.001), sensillum type (F_5,65_ = 396.40, p<0.001) and antenna per sensillum type interaction (F_5,65_ = 17.89, p<0.001). Separate analyses for each sensillum type revealed a significant right antenna dominance in the number of olfactory sensilla trichodea type A (F_1,13_ = 21.26, p<0.001); no significant antenna effects were found in the number of sensilla basiconica (F_1,13_ = 1.47, p = 0.247), sensilla coeloconica (F_1,13_ = 3.61, p = 0.08) and sensilla placodea (F_1,13_ = 0.97, p = 0.342). Analyses of non-olfactory sensilla did not reveal any significant difference between right and left antennae in the number of sensilla trichodea type B (F_1,13_ = 3.45, p = 0.086) and sensilla ampullacea (F_1,13_ = 0.10, p = 0.755).

## Discussion

The results extended previous findings on olfactory asymmetries in hymenopteran insects [11], [14], by showing a right side dominance in short-term recall of olfactory memory in another Apoidea species, *B. terrestris*. Bumble bees conditioned to extend their proboscis (PER) revealed better learning performance when trained with their right rather than their left antenna, with a magnitude comparable to that previously found in *A. mellifera* [11], [14].

In honey bees lateralization of olfactory learning is associated with morphological and electrophysiological asymmetries: the number of olfactory sensilla and the electroantennographic responses have been shown to be higher in the right than in the left antenna [11], [14], [16]. In the present study no significant overall differences in EAG responses between the right and the left antenna of bumble bees were observed. Nevertheless, there was a considerable trend when looking on the number of individuals showing significant lateralization (12 vs. 20), and among this subset of individuals the majority showed stronger responses with the right antenna (9 vs. 3). Since electroantennography records the sum of responses of all olfactory receptor neurons housed in the sensilla of a single antenna, the results obtained using SEM might explain the difference with the data obtained in honey bees. Only one class of bumble bee olfactory sensilla, trichodea type A, exhibited an anatomical asymmetry, being more abundant on the surface of the right antenna than on the left one, and a slight tendency emerged for a second class, i.e. sensilla coeloconica. On the other hand, sensilla placodea, the most common olfactory organs in Apoidea species, did not show any considerable asymmetrical distribution in *B. terrestris*. This can explain why no overall asymmetry was observed in EAG responses in bumble bees. Other factors may have also contributed to the species difference, i.e. the number of receptor neurons in each sensillum category and the number of receptor sites in each olfactory neuron, that could be independently associated with the gain or loss of asymmetry in the mechanisms of peripheral perception. The nematode *Caenorhabditis elegans* provides a striking example of the multiple factors contributing to lateralized odour detection in invertebrates. In this species it has been observed that a symmetrical distribution of olfactory sensory neurons hides an asymmetrical pattern on their surface of the G-protein-coupled olfactory receptors responsible for functional odour lateralization [4].

Kells and Goulson [26] noticed that three species of bumble bees, *Bombus lapidarius*, *Bombus lucorum* and *Bombus pascuorum*, showed preferences in the directions of circling when they visited florets arranged in circles around a vertical inflorescence. Interestingly, they did not observe any lateralization in *B. terrestris*. It could be that lateralization in circling is mainly due to antennal asymmetries (and not to higher level mechanisms associated with learning and memory recall). Even in honey bees the evidence suggests that peripheral asymmetries in receptors density and EAG antennal responses could not entirely account for asymmetries in memory recall as evinced from PER responses. Rogers and Vallortigara [13] showed that at 1–2 hour after training using both antennae, recall was possible only when the honey bees used their right antenna but by 6 hours after training the memory could be recalled only when the left antenna was in use. Clearly, asymmetries in receptor density could not account for this time-dependent shift in lateralization associated with memory consolidation [16].

It has been argued, however, that lateralization of function initially evolved in bilateral animals to increase brain efficiency [17]. Thus, morpho-physiological biases of the peripheral nervous system in insects could be a consequence of the embedded brain asymmetry [14]. Furthermore, the rates of olfactory lateralization in bumble bees evidenced for the first time in the present work corroborates the hypothesis of a link between levels of social interactions and the alignment of the direction of asymmetries in a population. Mathematical models of the evolution of population-level asymmetries based on game theory [17] pointed out that shared directionality in a population might arise as an evolutionary strategy driven by living in a social group, where individually asymmetrical organisms have to coordinate their behaviours with the behaviour of other conspecific individuals. The key concept would be that an individual within a social group benefits from acting according to the behaviour of the majority of the group individuals. According to the model the minority group (e.g. in this case bumble bees with better performance with the left antenna) would be maintained by frequency-dependent selection. Ghirlanda et al. [27] extended the mathematical model examining intraspecific interactions, with antagonistic-synergistic behaviours. They showed that the consistency of direction of asymmetries in a population should result from the most relevant of the two interactions, in term of fitness contribution. Populations with higher rates of synergistic interactions were shown to be more strongly lateralized in the same direction. Thus, the involvement of inter-individual interactions could have been a crucial factor for the evolution of lateralization in the olfactory associative learning also in *B. terrestris*. With respect to honey bees, the bumble bees annual society represents a less developed system in individuals exchanging information [19], [20]. They show a complete lack of both trophallaxis and transmission of definite geographical cues but the communication between colony members appears to play a key role in the nest as well. As a matter of fact, bumble bee foragers release from tergal gland a foraging recruitment pheromone that induces the inactive workers to leave the nest in search of food sources [28]. Moreover, a learning process of the currently rewarding floral odours occurs inside the nest driven by the olfactory information flow carried on the successful incoming bees in the honey pots; it has been suggested that the inter-individual contacts significantly improve odour learning and foragers recruitment [29], [30].

In conclusion, the data described here add to increasing evidence that lateralization of the nervous system is common in invertebrate species. Likewise, our findings on strictly related Apoidea species with different forms of social organization may confirm altogether the hypothesis of the relationship between social behaviour and the evolution of population-level asymmetries also in arthropods. Future studies on other species of bumble bees (*Bombus* spp.), or other Apoidea species characterized by different social or pre-social behaviours, such as gregarism, may provide additional insights to understand how strategic inter-individual interactions in a population have been powerful forces in the evolution of asymmetries.
